# Accelerated regrowth of non-small-cell lung tumours after induction chemotherapy

**DOI:** 10.1038/sj.bjc.6601418

**Published:** 2003-12-09

**Authors:** S Y El Sharouni, H B Kal, J J Battermann

**Affiliations:** 1 Department of Radiation Oncology Q00.118, University Medical Centre, Post Box 85500, Heidelberglaan 100, 35 84 CX Utrecht, The Netherlands

**Keywords:** NSCLC, waiting time, accelerated proliferation, tumour doubling time, induction chemotherapy

## Abstract

Induction chemotherapy of non-small-cell lung cancer (NSCLC) stage III with gemcitabine and cisplatin for downstaging of the tumour with the aim for further treatment with ionising radiation is one of the treatments for lung cancer patients. The purpose of this study was to investigate the influence of the waiting time for radiotherapy, that is, the interval between induction chemotherapy and radiotherapy, on the rate of tumour growth for patients with NSCLC. Interval times between the end of induction chemotherapy and date of diagnostic CT, planning CT and first day of radiotherapy were determined for 23 patients with NSCLC. Increase in gross tumour volume was measured for 18 patients by measuring the dimensions of the primary tumour and lymph node metastases on the diagnostic CT after induction chemotherapy and on the CT used for radiotherapy planning. For each patient, the volume doubling time was calculated from the time interval between the two CTs and ratio of the gross volumes on planning CT and diagnostic CT. The mean time interval between end of chemotherapy and day of diagnostic CT was 16 days, and till first day of radiotherapy 80.3 (range 29 – 141) days. In all, 41% of potentially curable patients became incurable in the waiting period. The ratio of gross tumour volumes of the two CTs ranged from 1.1 to 81.8 and the tumour doubling times ranged from 8.3 to 171 days, with a mean value of 46 days and median value of 29 days. This is far less than the mean doubling time of NSCLC in untreated patients found in the literature. This study shows that in the time interval between the end of induction chemotherapy and the start of radiotherapy rapid tumour progression occurs as a result of accelerated tumour cell proliferation: mean tumour doubling times are much shorter than those in not treated tumours. As a consequence, the gain obtained with induction chemotherapy with regard to volume reduction was lost in the waiting time for radiotherapy. We recommend diminishing the time interval between chemo- and radiotherapy to as short as possible.

Lung cancer is the leading cause of cancer death in both men (32%) and women (25%) (Perez and Brady, 1998). In the last decades, there was a sharp increase in the incidence of lung cancer (Storm *et al*, 1999; Teppo *et al*, 1999). About two-third of non-small-cell lung cancer (NSCLC) patients are diagnosed with distant disease, which restricts the option of radically intended treatment to less than one-third of patients (Jensen *et al*, 2002). The results of radiotherapy alone for lung cancer patients are still disappointing. Overall, only 5% of patients survived more than 5 years; locoregional control was about 20% after 5 years and more than 60% of patients developed distant metastases (Fietkau, 2001).

Shortening of the overall treatment time (OTT) improved local control and survival after radiotherapy of lung cancer patients (Fu *et al*, 1997; Bonner *et al*, 1998; Saunders *et al*, 1999), indicating the importance of cellular repopulation as a cause of failure in the radiotherapy of NSCLC (Saunders *et al*, 1997; Fowler and Chappell, 2000). Furthermore, tumour progression during the waiting time till the start of radiotherapy for lung cancer and head-and-neck tumours, respectively, was reported, indicating a possible negative influence on treatment results (O'Rourke and Edwards, 2000; Waaijer *et al*, 2003). Owing to restaging procedure after induction chemotherapy and waiting times for radiotherapy, we were interested to know to what extent the OTT and, in our case, the waiting period between the end of induction chemotherapy and the start of radiotherapy might influence tumour behaviour. To our knowledge, such a study on behaviour of NSCLC after induction chemotherapy has not been reported yet. The purpose of this study was to investigate the influence of the waiting time on the rate of tumour growth in patients with NSCLC treated with induction chemotherapy.

## MATERIALS AND METHODS

In the period 1999–2000, 23 patients with stage III NSCLC received induction chemotherapy with cisplatin and gemcitabine in the University Medical Centre Utrecht and in 10 regional hospitals. Gemcitabine was administered at a dose of 1000–1250 mg m^−2^ on days 1 and 8, and in some regional hospitals also on day 15. Cisplatin was given at doses ranging from 80 to 100 mg m^−2^ on day 1. The treatment was repeated every 3–4 weeks. In general, the 23 patients received 3–4 cycles before re-evaluation with CT scan and 22 were referred to the Radiotherapy Department in Utrecht with curative intent for stages III-A and downstaged III-B NSCLC. Patient characteristics – gender, age and histology, curative or palliative intent – are shown in Table 1

**Table 1:** Patient characteristics

*Gender*	
Male	13
Female	10
	
*Age (years)*	
Mean	59.3
Range	41–73
	
*Histology*	
Squamous cell carcinoma	7
Adenocarcinoma	3
Large-cell carcinoma	9
Not defined	4
	
*Referral to Radiotherapy Department*	
Curative intent	22
Palliative intent	1
	
*Radiotherapy*	
Curative irradiation	13
Palliative irradiation	10

.

A retrospective study was performed to evaluate the duration of the waiting period between the end of induction chemotherapy and the start of radiotherapy, and to look at tumour volume increase in that waiting period. CT scans were made for re-evaluation of tumour response after induction chemotherapy (CTr) at the referring hospitals and for planning purposes (CTp) at our Radiotherapy Department. Before CT planning, contrast infusion was given to all patients. Most of the diagnostic CT and all planning CT scans were spiral scans. The diagnostic scans were performed with breath-hold, the planning CT's during quiet respiration. Tumour movement as a consequence of both cardiac and respiratory activity may occur with the greatest average movement near 1 cm (Ross *et al*, 1990). However, for the present analysis of tumour volumes and subsequent tumour volume doubling times, changes in organ positions would not significantly affect the analyses of changes in apparent physical volume due to the state of breathing at the time of CT data acquisition (Balter *et al*, 1996). The gross tumour volumes, that is, the sum of the volume of the primary tumour and that of a lymph node metastasis if present, *Vr* at restaging and *Vp* on planning CT, could be determined for 18 patients. The delineation of tumour volume on CT scans was performed using PLATO IPS version 2.7 (Nucletron, The Netherlands). Tumour volume *V* was calculated by multiplying 0.5 times the maximum diameters in ventral/dorsal *d*_vd_ and lateral directions *d*_l_ and the number *n* of CT slices in craniocaudal direction on which the tumour was visible times slice thickness *t*: *V*=0.5*d*_vd_*d*_l_*nt*. CTr scans had a slice thickness of 8–10 mm; CTp scans had a slice thickness of 5 mm. Measurements were performed by one observer (SYES) without the involvement of a radiologist. Most of the restaging CT's were made in the regional hospitals and we were not able to use the digital formats of these CT's. Therefore, all CT's for restaging and planning purposes were analysed using the same method as described. For each patient the gross tumour volumes *Vr* and *Vp* were calculated and with the time interval *T* between CTr and CTp, the tumour volume doubling time *Td* could be estimated: *Td*=*T*ln2*/*ln(*Vp/Vr*) (Hasegawa *et al*, 2000).

According to our protocol, patients with stage III-B NSCLC receive palliative radiotherapy and with stage III-A high-dose radiotherapy with curative intent. In case of downstaging from stage III-B to III-A or no upgrading from stage III-A to III-B high-dose locoregional radiotherapy was given. Otherwise, palliative radiotherapy was given. The given doses for curative intended radiotherapy was 66 Gy in 33 fractions, 5 times/week, and for palliative radiotherapy it was 30 Gy in 10 fractions of 3 Gy in 4 fractions/week.

## RESULTS

After induction chemotherapy, 23 patients were referred to the Radiotherapy Department. One patient had complete response and 17 patients had partial response, thus the response rate after chemotherapy was 78% (18 out of 23 patients). In all, 22 patients were referred for treatment with curative intent. However, nine out of these 22 patients (41%) had progression of their disease in the waiting period to such extent (i.e. upgrading to stage III-B) that they became ineligible for high-dose radiotherapy. The interval time between the end of induction chemotherapy and CTr was 15.8 days (range −14 to 33 days; one patient had CTr during chemotherapy). The interval between CTr and CTp was 52.3 days (range 16–99 days), and interval between end of chemotherapy and first day of radiotherapy was 80.3 days (range 29–141 days). The overall treatment time, from the start of the chemotherapy till the end of radiotherapy varied between 115 and 219 days, Table 2

**Table 2:** Mean duration of treatments and interval times with range (day)

Induction chemotherapy	59.6 (37–98)
Interval end of chemotherapy – CTr	15.8 (−14^a^–33)
Interval CTr – CTp	52.3 (16–99)
Interval end of chemotherapy – 1st consultation radiotherapist	46.0 (1–80)
Interval 1st consultation – CTp	15.7 (11–40)
Interval CTp – 1st irradiation	14.1 (6–20)
Interval end of chemotherapy – 1st irradiation	80.3 (29–141)
*Radiotherapy*	
Curative intent	44.3 (30–50)
Palliative intent	11.1 (8–13)
	
Total treatment time	169.8 (115–219)

a One patient had CT for restaging 2 weeks before the end of induction chemotherapy.

. Based on CTr and CTp scans, all patients had tumour volume increase. Gross tumour volumes at CTr varied between 1 and 367 cm^3^, at the moment of CTp they varied between 45 and 793 cm^3^, Table 3

**Table 3:** The interval between CTr and CTp, gross tumour volumes at CTr and CTp, tumour volume doubling time Td, number of Td's in waiting period (i.e. the end of chemotherapy, the start of radiotherapy) and ratio of gross tumour volumes

**Patient no.**	**Interval CTr–CTp (days)**	**Gross tumour volume at CTr (cm^3^)**	**Gross tumour volume at CTp (cm^3^)**	**Td (days)**	**Waiting period (days)**	**Number of Td in waiting period**	**Volume at CTp/ volume at CTr**
4	68	14	793.5	11.7	106	9.1	56.7
5	88	62	112.9	101.7	101	1	1.8
6	49	26.3	99.2	25.6	102	4	3.8
7	38	9.9	57.2	15	62	4.1	5.8
8	99	51.7	600.7	28	141	5	11.6
9	53	1	81.8	8.3	83	10	81.8
10	48	25.5	51.8	46.9	72	1.5	2
12	44	9.6	48.5	18.8	64	3.4	5.1
13	16	242.4	258.6	171.4	49	0.3	1.1
14	42	85	223	30.2	62	2.1	2.6
15	71	48	104.1	63.6	68	1.1	2.2
16	36	25.2	60.3	28.6	77	2.7	2.4
17	27	36	60.1	36.5	29	0.8	1.7
18	25	367.4	752	24.2	63	2.6	2
20	57	91	298.5	33.3	91	2.7	3.3
21	85	160	253.9	127.6	76	0.6	1.6
22	48	18.8	45.2	37.9	108	2.8	2.4
23	48	15.75	127.2	15.9	91	5.7	8.1
							
Mean		71.6	223.8	45.8		3.3	10.9
							
Median		31.2	108.5	29.4		2.7	2.5

**Figure 1:**
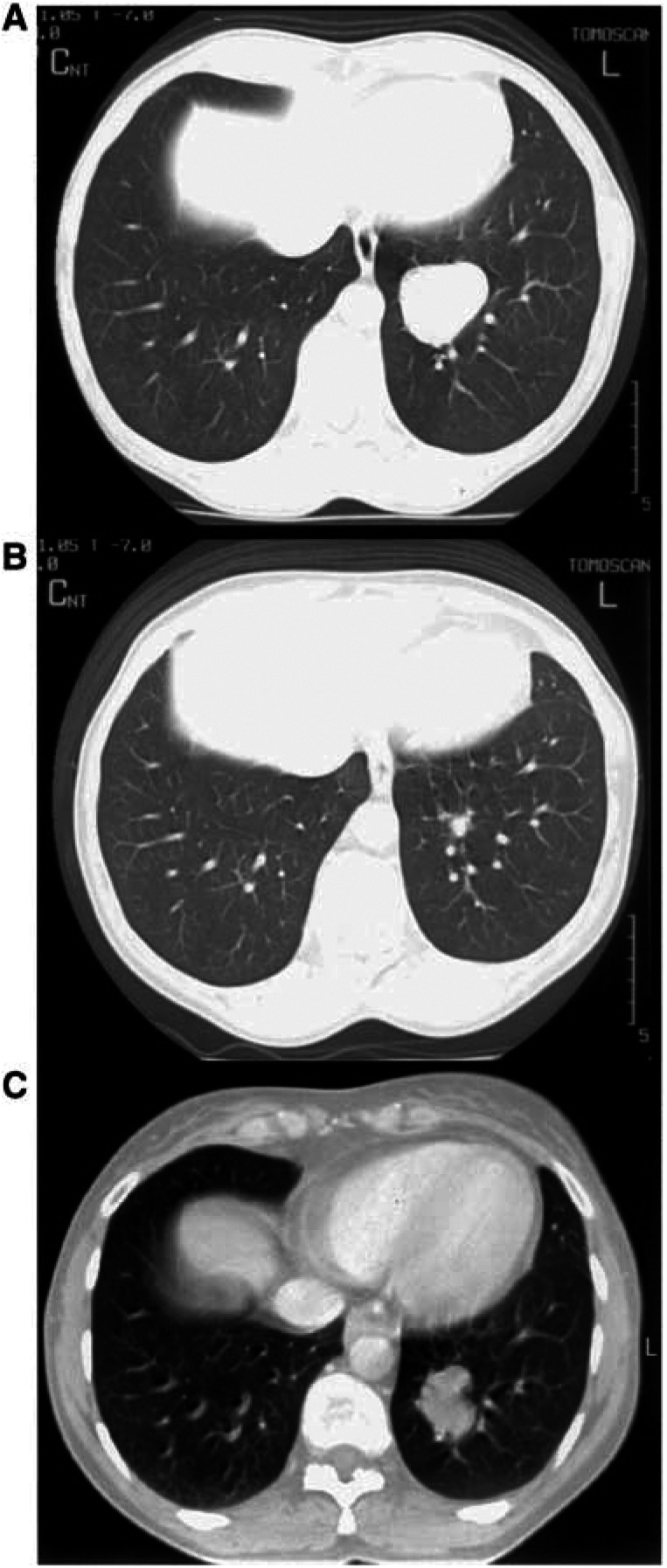
(**A**) CT scan of a NSCLC 78 days before induction chemotherapy; (**B**) CT scan made 55 days after the start of induction chemotherapy with gemcitabine and cisplatin; and (**C**): CT scan of the same tumour 72 days after induction chemotherapy.

**Figure 2:**
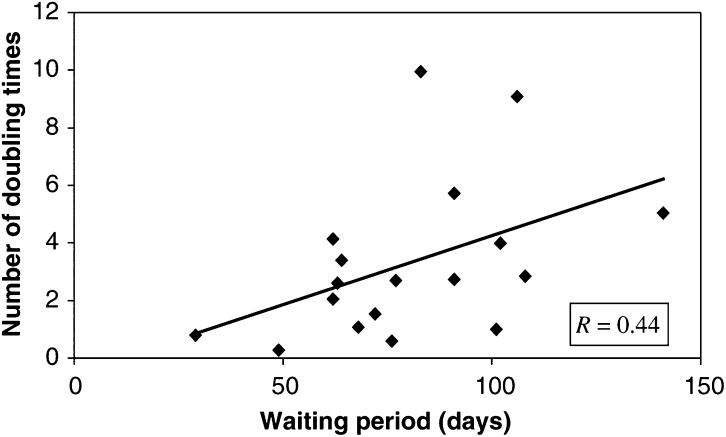
The number of doubling times in the waiting period between the end of induction chemotherapy and the start of radiotherapy as a function of waiting period.

. For the patient with complete response, the volume at CTr could not be determined, the volume was assumed to be 1 cm^3^. In Figure 1

, the CTs of a patient made 78 days before the induction chemotherapy, 55 days after the start of chemotherapy and 72 days after the end of chemotherapy for planning purposes, are shown. It illustrates the efficacy of the induction chemotherapy and the fast regrowth of the tumour after chemotherapy. The ratios of gross tumour volumes at CTp and at CTr are shown in Table 3. It varies from 1.1 to 81.8. The Td values are shown in Table 3. Td values ranged from 8.3 to 171.4 days with a mean of 45.8 days and a median value of 29.4 days. The number of tumour volume doubling times in the waiting period between the end of induction chemotherapy and the start of radiotherapy was calculated by dividing waiting time by tumour volume doubling time, Table 3. The number of Td's as a function of the waiting period is also presented in Figure 2

. Although the correlation coefficient is rather low, it demonstrates that the number of Td's increases for longer waiting periods. The number of Td's in the waiting period ranges from 0.3 to 10, the mean is 3.3 and the median value is 2.7.

**Figure 3:**
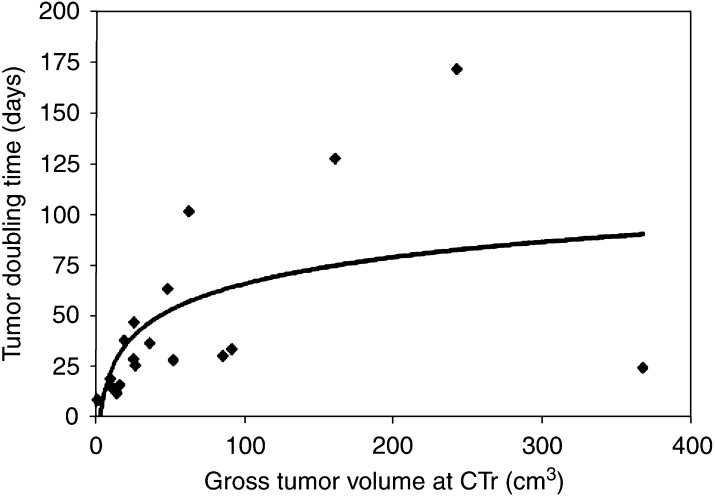
Tumour volume doubling time of gross tumour as a function of volume at CT for restaging.

The tumour doubling times as a function of the volume as determined with CTr (starting volume) are shown in Figure 3

. It illustrates that the tumours with the smallest starting volumes after chemotherapy had the fastest Td, indicating fast proliferating of the tumour cells surviving the induction chemotherapy.

## DISCUSSION

### Waiting time

In the last years, delays in starting radiotherapy is becoming an increasing problem. Apart from the psychological distress for the patients, the question is whether waiting times and delays have any bearing on prognosis and treatment. Specifically, the hypothesis is raised that longer delays are associated with poorer survival or more advanced stage disease. A strong independent association between tumour volume and survival in patients with NSCLC was reported (Etiz *et al*, 2002; Bradley *et al*, 2002; Willner *et al*, 2002). It was recommended that waiting times for radiotherapy should be as short as reasonably achievable (ASARA) (Mackillop *et al*, 1996). Delay in treatment increases the risk that metastases will develop before treatment is started. Treatment delay may also lead to increased complication rate. As tumours increase in size, larger volumes of normal tissue have to be irradiated to encompass them, and the probability of radiation complications increases as a function of the volume irradiated.

O'Rourke and Edwards (2000) described that in the waiting period for potentially curative radiotherapy that lasted from 35 to 187 days, six of their 29 lung cancer patients (21%) became incurable. An even larger percentage of patients in our study became incurable, nine of 22 potentially curable patients (41%) were treated with palliative intent after a waiting period ranging from 29 to 141 days. These nine patients had progression of their tumour to stage III-B at the time of planning CT and became ineligible for high-dose radiotherapy.

Waaijer *et al* (2003) investigated tumour growth of oropharyngeal tumours in the waiting time for radiotherapy and estimated an average control loss of 16–19% for these tumours during the waiting time.

Fortin *et al* (2002) concluded that delaying radiotherapy had a deleterious effect on patients with early head-and-neck squamous cell carcinomas. Radiotherapy should be started as soon as possible, preferably within 20–30 days after evaluation by a radiation oncologist.

Among patients with an upper aerodigestive tract cancer, professional delays of more than 1 month contributed to an increased risk for being diagnosed with late-stage disease (Allison *et al*, 1998). However, no significant correlation between waiting time and the outcome of early-stage laryngeal and nasopharyngeal cancers was found (Barton *et al*, 1997; Brouha *et al*, 2000). Lee *et al* (1993) however, have shown that advanced stage of head-and-neck tumours have a clear negative effect on treatment results. From the above reports, we conclude that long waiting times and delays may lead to important deterioration in local control rates.

In the present study, we observed a large variety in waiting times for radiotherapy after induction chemotherapy varying from 29 to 141 days and an increase in tumour volume in all patients. Pulmonologists and radiotherapists made the decision for combined chemo/radiotherapy for NSCLC patients in our region; however, patients were referred only after postchemotherapy evaluation to the department of radiotherapy. The causes of the long waiting times, therefore, are the restaging procedure after the induction chemotherapy, the time to overcome possible side effects of the chemotherapy, the time till referring patients as well as the waiting time from referring the patient till the start of radiotherapy (waiting time for the first visit, for performing the planning CT and for the start of radiotherapy). In that waiting period, we observed an increase in the gross tumour volume with a factor of more than 3. This volume increase, however, is faster after induction chemotherapy than in untreated tumours.

### Repopulation and tumour doubling time

There are many publications on experimental tumours that have shown rates of repopulation after radiotherapy that are equal to or often faster than the rates of cell repopulation in tumours without radiotherapy (Hermens and Barendsen, 1967; Suit and Urano, 1969; Abe *et al*, 1991; Begg *et al*, 1991; Milas *et al*, 1994). Intervals between chemotherapy doses are needed to allow repopulation of normal tissues. During these intervals, however, the surviving tumour cells can proliferate and repopulate (Stephens and Peacock, 1977; Rosenblum *et al*, 1976; 1983; Milas *et al*, 1994).

Data on tumour volume doubling time (Td) for human lung tumours are reported by Hasegawa *et al* (2000), Steel (1977), Usuda *et al* (1994), Fujimura *et al* (1979), Filderman *et al* (1986) and Geddes (1979). The data are summarised in Table 4

**Table 4:** Mean tumour volume doubling times (Td's) as reported in the literature and mean Td of the present study.

**Reference**	**Tumour type**	**Mean Td (days)**	**Overall mean Td (days)**
Hasegawa *et al* (2000)	Adenocarcinoma	533	
	Squamous cell carcinoma	129	
			452
Steel (1977)	Adenocarcinoma	148	
	Squamous cell carcinoma	85	
	Undiffer. tumours	79	
			104
Usada *et al* (1994)	Adenocarcinoma	163	
	Squamous cell carcinoma	80	
	Large-cell carcinoma	67	
			103
Fujimura *et al* (1979)	Adenocarcinoma	116	
	Large-cell carcinoma	71	
			93
Filderman *et al* (1986)	Adenocarcinoma	180	
	Large-cell carcinoma	100	
			140
Geddes (1979)	Adenocarcinoma	161	
	Squamous cell carcinoma	88	
	Large-cell carcinoma	86	
			102
Present results			46

. They indicate that for untreated NSCL tumours the mean Td is in excess of 93 days. O'Rourke and Edwards (2000) reported that the delay between diagnostic CT scan and planning CT amounted 18–131 days with a median of 54 days. Tumour growth in terms of percentage change in tumour cross-sectional area ranged from 0 to 373% with a median increase of 19%. If this value of 19% is used for the median interval of 54 days, a Td of 68 days can be derived, and for an interval of 113 days, the Td is 143 days.

In the present study, we observed after induction chemotherapy a clear progression in tumour volume with Td's varying from 8.3 to 171.4 days with a mean and median value of 45.8 and 29.4 days, respectively. This latter Td value is far less than Td's found for untreated NSCLC, Table 4. It indicates accelerated repopulation of cells surviving the induction chemotherapy course. Our findings are in line with those of others who observed a rapid regrowth after irradiation of pulmonary metastases (Battermann *et al*, 1981), and after surgery in head-and-neck cancer (Trotti *et al*, 1998; Ang *et al*, 2001; Awwad *et al*, 2002). In their review, Davis and Tannock (2000) reported on repopulation of tumour cells between cycles of chemotherapy as a neglected factor. We can conclude that fast regrowth of remaining tumour cells occurs after induction chemotherapy, radiotherapy and surgery.

As illustrated in Figure 2 and Table 3, the number of Td's in the waiting period ranges from 0.3 to 10. The mean number is 3.3. Thus, in the waiting period the tumour volume increases with a factor of more than 3.

As shown in Figure 3, the small tumours have the shortest Td. For instance, tumours with a volume up to 40 cm^3^ have a mean Td of 24.5 days (range 8.3–46.9 days). To our knowledge such a short mean Td value for lung tumours has not been reported earlier. Hasegawa *et al* (2000) determined growth rate of small lung cancers detected on mass CT screening. The shortest Td they found was 52 days. From the CT scans inserted in Figure 1 in the paper by O'Rourke and Edwards (2000), a Td of 18.3 days can be derived. This value is for a patient receiving prior chemotherapy (O'Rourke, pers. com., 2003) and confirms our findings that after induction chemotherapy fast regrowth occurs. In our study, the shortest Td was 8.3 days.

*In conclusion*, we present evidence that after induction chemotherapy fast regrowth of NSCLC occurs and that accelerated repopulation of surviving tumour cells is responsible for the fast regrowth. It is clear that the beneficial result of induction chemotherapy, that is, tumour volume regression, has faded away. The Td is far shorter than that of untreated lung tumours. This influences the treatment results significantly. It is tragically that interval times up to more than 3 months are found whereas radiotherapy can be started within 1 month after induction chemotherapy as observed here. In all, 41% of potentially curable patients became incurable in that waiting period. We recommend that radiotherapy should start as soon as possible, preferably within 2–3 weeks, after the last chemotherapy cycle. Owing to the accelerated cell proliferation observed, accelerated radiotherapy should be given serious consideration to keep overall treatment time short. In further studies, concurrent chemo/radiotherapy treatment should be considered since a growing body of data show that concurrent chemo/radiotherapy improves survival in selected patients in stage III NSCLC (Schaake-Koning *et al*, 1994; Furuse *et al*, 1999; Curran *et al*, 2003).

## References

[bib1] Abe Y, Urano M, Kenton LA, Kahn J, Willet CG (1991) The accelerated repopulation of a murine fibrosarcoma, FSA-II, during the fractionated irradiation and the linear-quadratic model. Int J Radiat Oncol Biol Phys 21: 1529–1534 1938563 10.1016/0360-3016(91)90329-3

[bib2] Allison P, Franco E, Black M, Feine J (1998) The role of professional diagnostic delays in the prognosis of upper aerodigestive tract carcinoma. Oral Oncol 34: 147–153 9682779 10.1016/s1368-8375(97)00088-2

[bib3] Ang KK, Trotti A, Brown BW, Garden AS, Foote RL, Morrison WH, Geara FB, Klotch DW, Goepfert H, Peters LJ (2001) Randomized trial addressing risk features and time factors of surgery plus radiotherapy in advanced head-and-neck cancer. Int J Radiat Oncol Biol Phys 51: 571–578 11597795 10.1016/s0360-3016(01)01690-x

[bib4] Awwad HK, Lotayef M, Shouman T, Begg AC, Wilson G, Bentzen SM, Abd El-Moneim H, Eissa S (2002) Accelerated hyperfractionation (AHF) compared to conventional fractionation (CF) in the postoperative radiotherapy of locally advanced head and neck cancer: influence of proliferation. Br J Cancer 86: 517–523 11870530 10.1038/sj.bjc.6600119 PMC2375281

[bib5] Balter JM, Ten Haken RK, Lawrence TS, Lam KL, Robertson JM (1996) Uncertainties in CT-based radiation therapy treatment planning associated with patient breathing. Int J Radiat Oncol Biol Phys 36: 167–174 8823272 10.1016/s0360-3016(96)00275-1

[bib6] Barton MB, Morgan G, Smee R, Tiver KW, Hamilton C, Gebski V (1997) Does waiting time affect the outcome of larynx cancer treated by radiotherapy? Radiother Oncol 44: 137–141 9288841 10.1016/s0167-8140(97)00093-5

[bib7] Battermann JJ, Breur K, Hart GA, van Peperzeel HA (1981) Observations on pulmonary metastases in patients after single doses and multiple fractions of fast neutrons and cobalt-60 gamma rays. Eur J Cancer 17: 539–548 7297593 10.1016/0014-2964(81)90056-6

[bib8] Begg AC, Hofland I, Kummermehr J (1991) Tumour cell repopulation during fractionated radiotherapy: correlation between flow cytometric and radiobiological data in three murine tumours. Eur J Cancer 27: 537–543 1828958 10.1016/0277-5379(91)90211-u

[bib9] Bonner JA, McGinnis WL, Stella PJ, Marschke Jr F, Sloan JA, Shaw EG, Mailliard JA, Creagan ET, Ahuja RK, Johnson PA (1998) The possible advantage of hyperfractionated thoracic radiotherapy in the treatment of locally advanced non-small cell lung carcinoma: results of a North Central Cancer Treatment Group Phase III Study. Cancer 82: 1037–1048 9506347

[bib10] Bradley JD, Ieumwananonthachai N, Purdy JA, Wasserman TH, Lockett MA, Graham MV, Perez CA (2002) Gross tumor volume, critical prognostic factor in patients treated with three-dimensional conformal radiation therapy for non-small-cell lung carcinoma. Int J Radiat Oncol Biol Phys 52: 49–57 11777621 10.1016/s0360-3016(01)01772-2

[bib11] Brouha XD, Op de Coul B, Terhaard CH, Hordijk GJ (2000) Does waiting time for radiotherapy affect local control of T1N0M0 glottic laryngeal carcinoma? Clin Otolaryngol 25: 215–218 10944052 10.1046/j.1365-2273.2000.00347.x

[bib12] Curran WJ, Scott CB, Langer CJ, Komaki R, Lee JS, Hauser S, Movsas B, Wasserman T, Sause W, Cox JD (2003) Long-term benefit is observed in a phase III comparison of sequential *vs* concurrent chemo-radiation for patients with unresected stage III non-small cell lung cancer: RTOG 9410. Annual Meeting of the American Society of Clinical Oncology May 31–June 3, Chicago (abstract 2499)

[bib13] Davis AJ, Tannock JF (2000) Repopulation of tumour cells between cycles of chemotherapy: a neglected factor. Lancet Oncol 1: 86–93 11905673 10.1016/s1470-2045(00)00019-x

[bib14] Etiz D, Marks LB, Zhou SM, Bentel GC, Clough R, Hernando ML, Lind PA (2002) Influence of tumor volume on survival in patients irradiated for non-small-cell lung cancer. Int J Radiat Oncol Biol Phys 53: 835–846 12095548 10.1016/s0360-3016(02)02814-6

[bib15] Fietkau R (2001) Concomitant radiochemotherapy of advanced non-small-cell lung cancer. Lung Cancer 33(Suppl. 1): S65–S76 11576710 10.1016/s0169-5002(01)00305-1

[bib16] Filderman AE, Shaw C, Matthay RA (1986) Lung cancer. Part I: etiology, pathology, natural history, manifestations, and diagnostic techniques. Invest Radiol. 21: 80–90 3003005 10.1097/00004424-198601000-00014

[bib17] Fortin A, Bairati I, Albert M, Moore L, Allard J, Couture C (2002) Effect of treatment delay on outcome of patients with early-stage head-and-neck carcinoma receiving radical radiotherapy. Int J Radiat Oncol Biol Phys 52: 929–936 11958885 10.1016/s0360-3016(01)02606-2

[bib18] Fowler JF, Chappell R (2000) Non-small cell lung tumors repopulate rapidly during radiation therapy. Int J Radiat Oncol Biol Phys 46: 516–517 10661362 10.1016/s0360-3016(99)00364-8

[bib19] Fu XL, Jiang GL, Wang LJ, Qian H, Fu S, Yie M, Kong FM, Zhao S, He SQ, Liu TF (1997) Hyperfractionated accelerated radiation therapy for non-small cell lung cancer: clinical phase I/II trial. Int J Radiat Oncol Biol Phys 39: 545–552 9336130 10.1016/s0360-3016(97)00332-5

[bib20] Fujimura S, Suda S, Yamauchi A, Sato H, Sohara Y, Kondo T (1979) Tumor doubling time and PPD skin test reactivity in resectable lung cancer. J Jpn Lung Cancer Soc 19: 135–142

[bib21] Furuse K, Fukuoka M, Kawahara M, Nishikawa H, Takada Y, Kudoh S, Katagami N, Ariyoshi Y (1999) Phase III study of concurrent versus sequential thoracic radiotherapy in combination with mitomycin, vindesine, and cisplatin in unresectable stage III non-small-cell lung cancer. J Clin Oncol 17: 2692–2699 10561343 10.1200/JCO.1999.17.9.2692

[bib22] Geddes DM (1979) The natural history of lung cancer: a review based on rates of tumour growth. Br J Dis Chest 73: 1–17 435370

[bib23] Hasegawa M, Sone S, Takashima S, Li F, Yang ZG, Maruyama Y, Watanabe T (2000) Growth rate of small lung cancers detected on mass CT screening. Br J Radiol 73: 1252–1259 11205667 10.1259/bjr.73.876.11205667

[bib24] Hermens AF, Barendsen GW (1967) Cellular proliferation patterns in an experimental rhabdomyosarcoma in the rat. Eur J Cancer 3: 361–369 6078936 10.1016/0014-2964(67)90020-5

[bib25] Jensen AR, Mainz J, Overgaard J (2002) Impact of delay on diagnosis and treatment of primary lung cancer. Acta Oncol 41: 147–152 12102158 10.1080/028418602753669517

[bib26] Lee WR, Mancuso AA, Saleh EM, Mendenhall WM, Parsons JT, Million RR (1993) Can pretreatment computed tomography findings predict local control in T3 squamous cell carcinoma of the glottic larynx treated with radiotherapy alone? Int J Radiat Oncol Biol Phys 25: 683–687 8454487 10.1016/0360-3016(93)90016-o

[bib27] Mackillop WJ, Bates JH, O'Sullivan B, Withers HR (1996) The effect of delay in treatment on local control by radiotherapy. Int J Radiat Oncol Biol Phys 34: 243–250 12118558 10.1016/0360-3016(95)02049-7

[bib28] Milas L, Nakayama T, Hunter N, Jones S, Lin TM, Yamada S, Thames H, Peters L (1994) Dynamics of tumor cell clonogen repopulation in a murine sarcoma treated with cyclophosphamide. Radiother Oncol 30: 247–253 8209009 10.1016/0167-8140(94)90465-0

[bib29] O'Rourke N, Edwards R (2000) Lung cancer treatment waiting times and tumour growth. Clin Oncol (R Coll Radiol) 12: 141–144 10942328 10.1053/clon.2000.9139

[bib30] Perez CA, Brady LW (1998) Principles and Practice of Radiation Oncology 3rd ed., Lippincot-Raven Publishers, Philadelphia–New York, USA. ISBN 0 397 58416 4

[bib31] Rosenblum ML, Gerosa MA, Dougherty DV, Wilson CB (1983) Improved treatment of a brain-tumor model Part. 1: advantages of single- over multiple-dose BCNU schedules. J Neurosurg 58: 177–182 6848673 10.3171/jns.1983.58.2.0177

[bib32] Rosenblum ML, Knebel KD, Vasquez DA, Wilson CB (1976) *In vivo* clonogenic tumor cell kinetics following 1,3-*bis*(2-chloroethyl)-1-nitrosourea brain tumor therapy. Cancer Res 36: 3718–3725 953998

[bib33] Ross CS, Hussey DH, Pennington EC, Stanford W, Doornbos JF (1990) Analysis of movement of intrathoracic neoplasms using ultrafast computerized tomography. Int J Radiat Oncol Biol Phys 18: 671–677 2318701 10.1016/0360-3016(90)90076-v

[bib34] Saunders M, Dische S, Barrett A, Harvey A, Gibson D, Parmar M (1997) Continuous hyperfractionated accelerated radiotherapy (CHART) versus conventional radiotherapy in non-small-cell lung cancer: a randomised multicentre trial. CHART Steering Committee. Lancet 350: 161–165 9250182 10.1016/s0140-6736(97)06305-8

[bib35] Saunders M, Dische S, Barrett A, Harvey A, Griffiths G, Palmar M (1999) Continuous, hyperfractionated, accelerated radiotherapy (CHART) versus conventional radiotherapy in non-small cell lung cancer: mature data from the randomised multicentre trial CHART Steering Committee. Radiother Oncol 52: 137–148 10577699 10.1016/s0167-8140(99)00087-0

[bib36] Schaake-Koning C, van den Bogaert W, Dalesio O, Festen J, Hoogenhout J, van Houtte P, Kirkpatrick A, Koolen M, Maat B, Nijs A, Renaud A, Rodrigus P, Schuster-Uitterhoeve L, Sculier J, van Zandwijk N, Bartelink H (1994) Radiosensitization by cytotoxic drugs. The EORTC experience by the Radiotherapy and Lung Cancer Cooperative Groups. Lung Cancer 10(Suppl. 1): S263–S270 8087519 10.1016/0169-5002(94)91690-x

[bib37] Steel GG (1977) The Growth Kinetics of Tumours. Oxford: Oxford University Press

[bib38] Stephens TC, Peacock JH (1977) Tumour volume response, initial cell kill and cellular repopulation in B16 melanoma treated with cyclophosphamide and 1-(2-chloroethyl)-3-cyclohexyl-1-nitrosourea. Br J Cancer 36: 313–321 921888 10.1038/bjc.1977.195 PMC2025422

[bib39] Storm HH, Dickman PW, Engeland A, Haldorsen T, Hakulinen T (1999) Do morphology and stage explain the inferior lung cancer survival in Denmark? Eur Respir J 13: 430–435 10065693 10.1183/09031936.99.13243099

[bib40] Suit H, Urano M (1969) Repair of sublethal radiation injury in hypoxic cells of a C3H mouse mammary carcinoma. Radiat Res 37: 423–434 5765551

[bib41] Teppo L, Dickman PW, Hakulinen T, Luostarinen T, Pukkala E, Sankila R, Soderman B (1999) Cancer patient survival – patterns, comparisons, trends – a population-based Cancer Registry study in Finland. Acta Oncol 38: 283–294 10380818 10.1080/028418699431348

[bib42] Trotti A, Klotch D, Endicott J, Ridley M, Cantor A (1998) Postoperative accelerated radiotherapy in high-risk squamous cell carcinoma of the head and neck: long-term results of a prospective trial. Head Neck 20: 119–123 9484942 10.1002/(sici)1097-0347(199803)20:2<119::aid-hed4>3.0.co;2-5

[bib43] Usuda K, Saito Y, Sagawa M, Sato M, Kanma K, Takahashi S, Endo C, Chen Y, Sakurada A, Fujimura S (1994) Tumor doubling time and prognostic assessment of patients with primary lung cancer. Cancer 74: 2239–2244 7922975 10.1002/1097-0142(19941015)74:8<2239::aid-cncr2820740806>3.0.co;2-p

[bib44] Waaijer A, Terhaard CH, Dehnad H, Hordijk GJ, van Leeuwen MS, Raaymakers CP, Lagendijk JJ (2003) Waiting times for radiotherapy: consequences of volume increase for the TCP in oropharyngeal carcinoma. Radiother Oncol 66: 271–276 12742266 10.1016/s0167-8140(03)00036-7

[bib45] Willner J, Baier K, Caragiani E, Tschammler A, Flentje M (2002) Dose, volume, and tumor control prediction in primary radiotherapy of non-small-cell lung cancer. Int J Radiat Oncol Biol Phys 52: 382–389 11872283 10.1016/s0360-3016(01)01823-5

